# Decitabine activates type I interferon signaling to inhibit p53‐deficient myeloid malignant cells

**DOI:** 10.1002/ctm2.593

**Published:** 2021-11-06

**Authors:** Jiale Wu, Yuntong Li, Jiaqi Wu, Huaxin Song, Yigang Tang, Ni Yan, Lili Wu, Sujiang Zhang, ChunKang Chang, Min Lu

**Affiliations:** ^1^ State Key Laboratory of Medical Genomics National Research Center for Translational Medicine (Shanghai) Shanghai Institute of Hematology Ruijin Hospital Affiliated to Shanghai Jiao Tong University School of Medicine Shanghai China; ^2^ Department of Hematology Shanghai Institute of Hematology Shanghai Jiao Tong University Affiliated Sixth People's Hospital Shanghai China

Dear Editor,

p53 is the most frequently mutated protein in cancers. We recently reported that arsenic trioxide (ATO) can efficiently restore tumour‐suppressor function to structural mutant p53,^1^, ^2^ spawning a series of p53‐targeted ATO clinical trials registered on *ClinicalTrials.gov*. However, ATO, together with other p53‐targeted agents, still lacks reports for statistically significant clinical efficacy at present. Targeting the molecular alterations caused by p53 mutations (for example, become addicted to a signaling), rather than directly targeting mutant p53 itself, is an alternative strategy to treat p53‐mutated patients.

We and others previously reported a high complete response rate of the decitabine (DAC) in treating p53‐mutated acute myeloid leukemia (AML) and myelodysplastic syndrome (MDS).^3^, ^4^, ^5^ This observation is particularly encouraging considering the high hazard ratio of *TP53* mutation for overall survival in myeloid malignancies (Figure S1A‐D). However, all responding p53‐mutated patients relapsed in a few months.^4^, ^5^ Thus, understanding mechanisms underlying the high response rate and the inevitable relapse is critical.

DAC and analogues were reported to trigger interferon (IFN) signaling in mouse embryonic fibroblasts (MEFs) and cancer cells.^6^, ^7^, ^8^ We confirmed the preferentially inhibition of cell growth and upregulation of IFN signaling genes by DAC in *Trp53* ^−/−^ MEFs (Figure S2A,B). Using isogenic myeloid malignant THP‐1 cells (originally null p53) infected with wild‐type (WT) p53, mutant p53 (R282W, a hotspot p53 mutation frequently detected in AML/MDS^5^) or vector (Figure S2C), we found that DAC also preferentially inhibited p53‐deficient THP‐1 lines (Figure S2D). In RNA‐seq, 5 µM DAC significantly upregulated 216 and 332 genes in p53‐R282W cells and p53 WT cells, respectively. The encoded proteins were highly enriched in an interaction network centering type I IFN signaling in p53‐R282W cells, but not in p53 WT cells (Figure 1A,B). Consistently, IFN signaling was the most enriched gene ontology biological process (GO BP) and reactome pathway in p53‐R282W cells, but not in p53 WT cells (Figure 1C,D).

**FIGURE 1:**
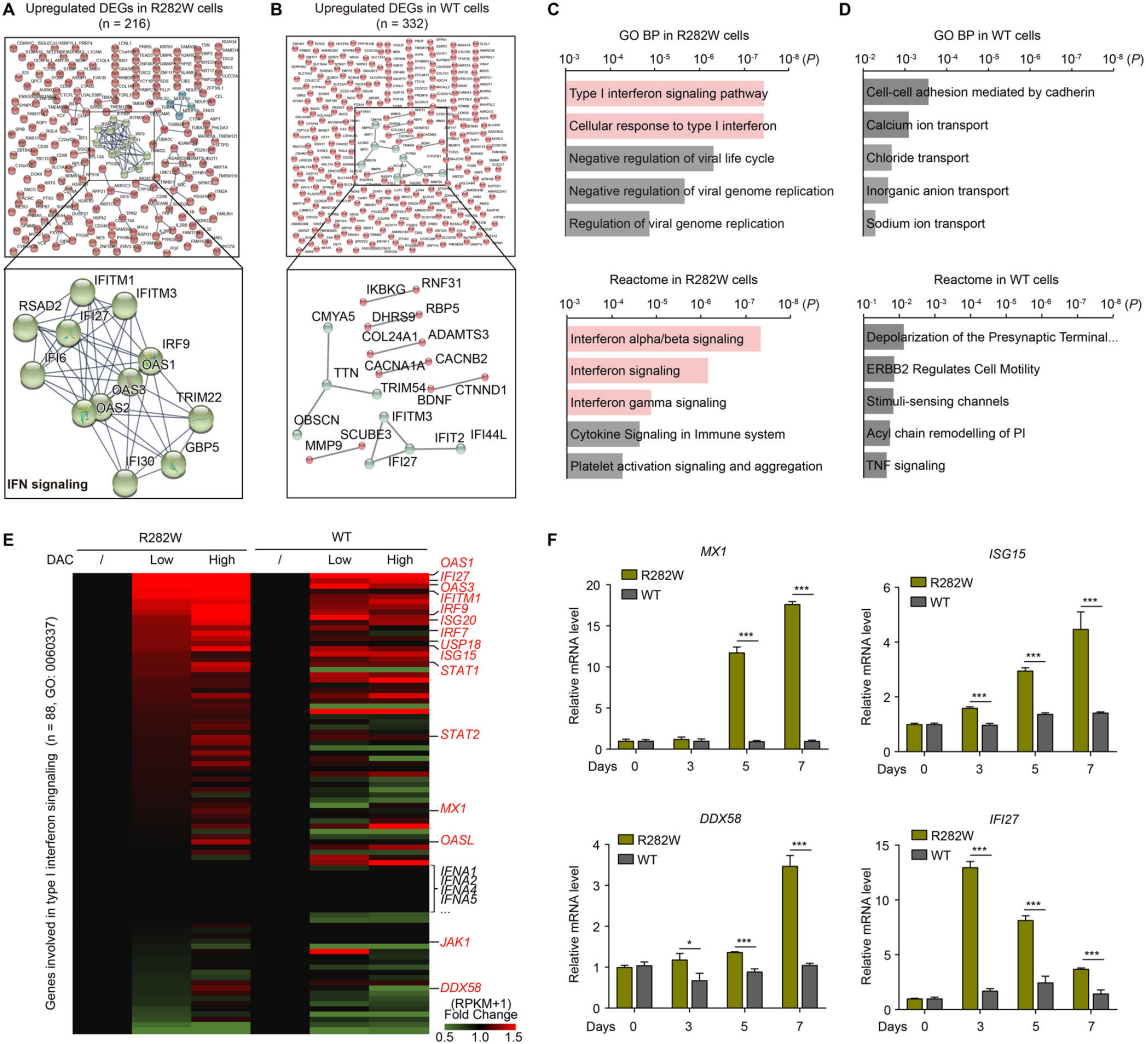
Decitabine (DAC) activates interferon (IFN) signaling to suppress the proliferation of p53‐deficient myeloid malignant cell lines. (A‐D) RNA‐seq analysis for the signaling activated by DAC in THP‐1 cells expressing p53‐R282W or wild‐type (WT) p53. Cells were treated with 1 µM or 5 µM DAC for 2 days, followed by ribonucleic acid (RNA) isolation and sequencing. Protein interaction for the 216 upregulated differentially expressed genes (DEGs) (Gfold ≥ 0.4, [reads per kilobase per million (RPKM) +1] fold change ≥ 1.5) in p53‐R282W cells (A) and the 332 upregulated DEGs in p53 WT cells (B) are shown. Enrichment assay for the upregulated DEGs in p53‐R282W cells (C) and p53 WT cells (D) by DAC. (E) Heatmap shows relative expression levels of type I IFN signaling genes in the DAC‐treated THP‐1 cells expressing p53‐R282W or WT p53. (F) Quantitative reverse transcription PCR (qRT‐PCR) determination for mRNA levels of the indicated genes in the cells treated with 0.2 µM DAC for 0, 3, 5 or 7 days, respectively. Error bars represent mean ± SD (*n* = 3, *p < 0.05, **p < 0.01, ***p < 0.001)

In the heatmap showing the relative expression levels of genes involved in type I IFN signaling, a batch of genes including *MX1*, *ISG15*, *DDX58* and *IFI27*, but not IFN alpha protein‐encoding genes (*IFNA1*, *IFNA2*, *IFNA4*, et al.), were efficiently upregulated following DAC treatment in p53‐R282W cells (Figure 1E). As expected, weaker upregulation was observed in p53 WT cells (Figure 1E). Upregulation of type I IFN genes was also observed in the myeloid malignant U937 cells expressing p53‐R282W upon DAC treatment (Figure S2E). The RNA‐seq results were confirmed by qRT‐PCR (Figure 1F).

The striking activation of IFN signaling by DAC in p53‐deficient THP‐1 cells can be achieved by upregulation of viral double strand RNAs (dsRNAs)^6^, ^7^, ^8^ or direct DNA demethylation of promoters of IFN signaling genes (Figure 2A). In qRT‐PCR, we observed upregulation of the representative dsRNAs *MER57B1* and *ERV3* (Figure 2B). In methylation‐specific PCR (MSP), the promoter of *IFNB1*, but not *IRF7* or *IFNA1*, was demethylated upon DAC treatment (Figure 2C). Thus, both mechanisms contribute to the observed activation of IFN signaling (Figure 2A).

**FIGURE 2:**
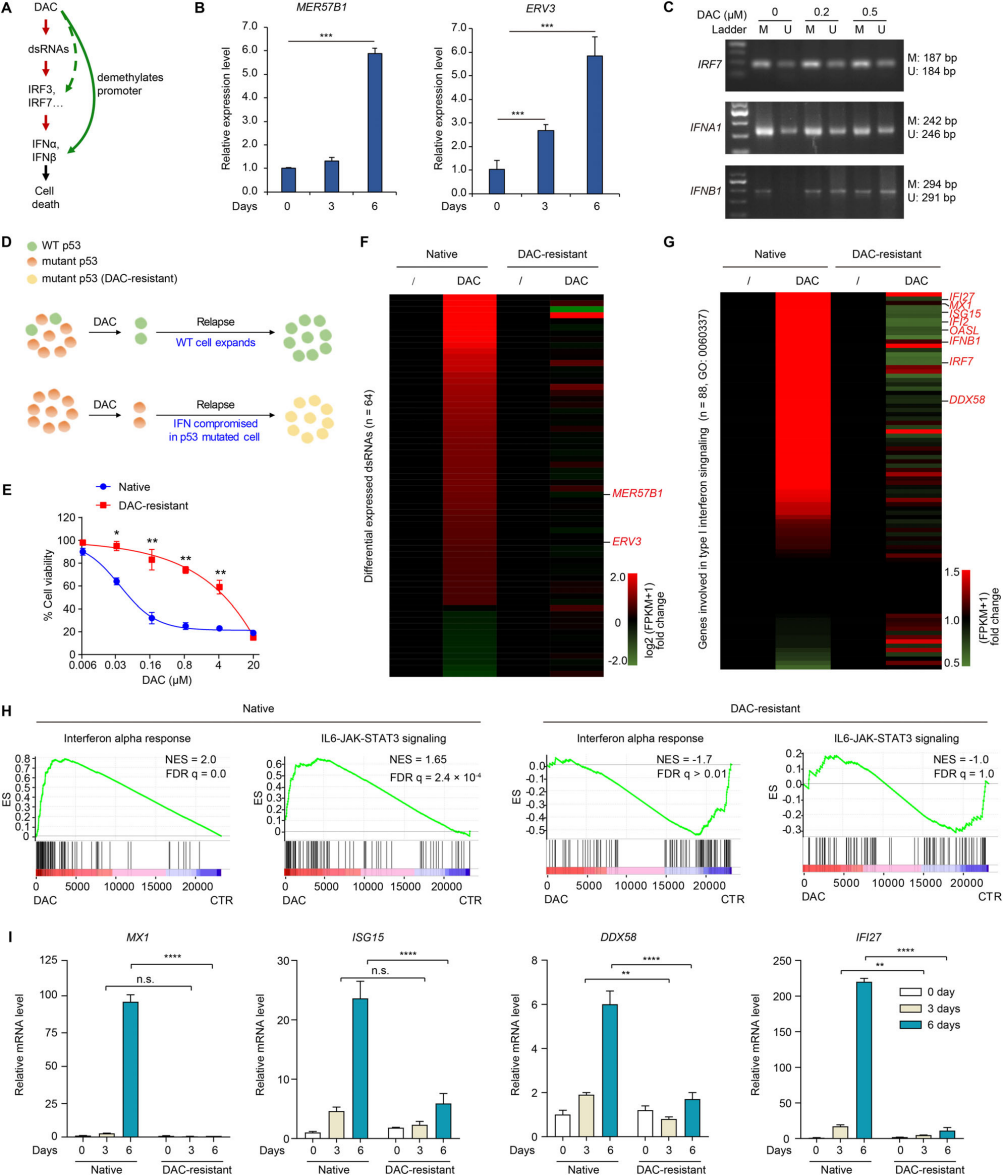
Decitabine (DAC) failed to activate interferon (IFN) signaling in DAC‐resistant THP‐1 cell line. (A) The potential mechanisms of IFN signaling activation by DAC in p53‐deficient THP‐1 cells. (B) qRT‐PCR determination for expression levels of the representative dsRNAs in THP‐1 cells upon 0.2 µM DAC treatment for 0–6 days. (C) Methylation‐specific PCR detecting the promoters of *IRF7*, *IFNA1* and *IFNB1* genes in THP‐1 cells treated with indicated concentration of DAC for 6 days. U, unmethylated alleles; M, methylated alleles. (D) The potential mechanisms of DAC resistance. (E) Cell viability of native and DAC‐resistant THP‐1 cells upon DAC treatment for 3 days. (F and G) Heatmap shows relative expression levels of differential expressed dsRNAs (log2 [fold change] > 1 and *p* < 0.05 in DAC‐treated cells compared to untreated cells) (F) and type I IFN signaling genes (G) in native and DAC‐resistant THP‐1 cells upon DAC treatment. (H) Gene set enrichment analysis (GSEA) for the transcriptomes of DAC‐treated cells with normalized enrichment scores (NESs) and false discovery rate (FDR) q values shown. (I) qRT‐PCR determination for mRNA levels of the indicated genes in the native and DAC‐resistant THP‐1 cells upon 1 µM DAC treatment for 0–6 days. Error bars represent mean ± SD (*n* = 3, *p < 0.05, **p < 0.01, ***p < 0.001, ****p < 0.0001)

We further explored two potential mechanisms underlying DAC resistance (Figure 2D). The expanding of pre‐existing p53‐mutated subclones in DAC relapsed patients supported the second mechanism.^5^ We thus prepared DAC‐resistant THP‐1 cells by long‐term DAC treatment (Figures S3A,B and 2E). RNA‐seq results revealed that the DAC‐mediated upregulations of dsRNAs and type I IFN signaling genes were largely compromised in DAC‐resistant cells (Figure 2F‐H). The RNA‐seq results were confirmed by qRT‐PCR (Figure 2I).

We next tried to generate a mouse model of p53‐deficient AML for in vivo studies. The p53‐deficient mice (*Trp53* ^−/−^) did not develop AML over their lifespan (Figure 3A). In contrast, the mice transplanted with *Trp53* ^−/−^; *Kras* ^G12D^ fetal liver cells developed lethal neoplasia on day 20 in both of the first and second transplantations (Figure 3B,C). Immature blast cells were clearly detected in the peripheral blood (PB) and bone marrow (BM) at day 7 upon injection of *Trp53* ^−/−^; *Kras* ^G12D^ fetal liver cells (Figure 3D). Hepatomegaly, splenomegaly, anaemia and reduced platelet counts were also observed (Figure S4A,B). Staining of the isolated BM, PB and spleen (SP) using myeloid (Figure S4C), lymphoid (Figure S4D), T‐cell (Figure S4D), erythrocyte (Figure 3E), hematopoietic stem cell and hematopoietic progenitor cell lineage markers (Figure S4E) suggested that an erythrocyte‐abnormal AML mouse model was successfully generated (Figure 3B, lower panel).

**FIGURE 3:**
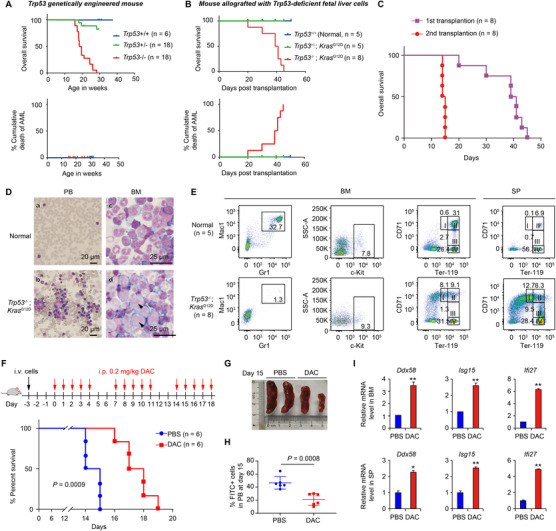
Decitabine (DAC) activates interferon (IFN) signaling in a mouse model of p53‐deficient acute myeloid leukemia (AML). (A) Kaplan–Meier survival curves (upper) and AML cumulative death curve (lower) of *Trp53* ^−/−^, *Trp53* ^+/−^ and *Trp53* ^+/+^ genetically engineered mice. (B) *Trp53* ^+/−^ or *Trp53* ^−/−^ fetal liver cells infected with *Kras* ^G12D^ were transplanted into lethally irradiated mice. Kaplan–Meier survival curves (upper) and AML cumulative death curve (lower) are shown. (C) Second transplantation of bone marrow (BM) from moribund AML mice into sub‐lethally irradiated mice. Kaplan–Meier survival curve is shown. (D) Representative histopathology of peripheral blood (PB) and BM derived from *Trp53* ^−/−^; *Kras* ^G12D^ mice and age‐matched normal mice. Immature blast cells are labelled with arrowheads. (E) Representative flow cytometry profiles of BM and spleens (SPs) derived from the indicated mice. The CD71 and Ter‐119 markers for erythrocytes were stained. (F) BM from moribund AML mice were transplanted into sub‐lethally irradiated mice on day −3. Mice were treated with phosphate buffered saline (PBS), 0.2 mg kg^−1^ DAC intraperitoneally (i.p.) for 5 consecutive days per week from day 0. Kaplan–Meier comparative survival curve is shown. (G) Representative spleens dissected from mice that died of AML at day 15 in the untreated group and the matched‐day mice in the DAC group. (H) Flow cytometry analysis of the rates of GFP positive cells (the transplanted AML cells) in PB at day 15 in the untreated group and the matched‐day mice in the DAC group. (I) qRT‐PCR determination of the indicated genes in the BM and PB cells derived from indicated groups of mice at day 15. Error bars represent mean ± SD (*n* = 3, *p < 0.05, **p < 0.01)

The established AML mice were then intraperitoneally injected with 0.2 mg kg^−1^ DAC or PBS for 5 consecutive days each week (Figure 3F). Mice in the DAC‐treated group survived significantly longer than the untreated group, with median survival increasing from 14.5 to 17.5 days (Figure 3F, *p* = 0.0009). In a parallel experiment, the spleens at day 15 in the DAC‐treated group were generally smaller (Figure 3G), and the percentages of AML cells in the spleens were significantly lower (Figure 3H). In the BM and SP, the mRNA levels of IFN signaling genes were significantly upregulated in the DAC group (Figure 3I), which were consistent with the finding in Figure 2I.

In our AML/MDS Sample Bank at the Shanghai Institute of Hematology, five BM and PB samples were recorded as p53‐mutated at prognosis (Figure 4A), with the samples from patients #4 and #5 receiving DAC treatment but experiencing disease progression at the time of sample collection. We confirmed these p53 mutants were functionally deficient by the classical luciferase reporter assay^9^ (Figure 4B). The cryopreserved primary BM mononuclear cells (BMMC) or PBMCs were thawed and cultured for 3 days with or without DAC. The current culture system did not greatly cause PBMCs death (Figure S5A,B). Upon DAC treatment for 3 days, the mRNA levels of the representative IFN signaling genes were consistently upregulated in the cultured primary cells from patients #1, #2 and #3, with *IFI27* efficiently upregulated (Figure 4C). By contrast, we did not observe significant upregulation of these IFN signaling genes in the samples derived from patients #4 and #5 with DAC relapse (Figure 4C). We failed to perform additional experiments on this set of primary cells such as RNA‐seq because of the limited cell amounts.

**FIGURE 4:**
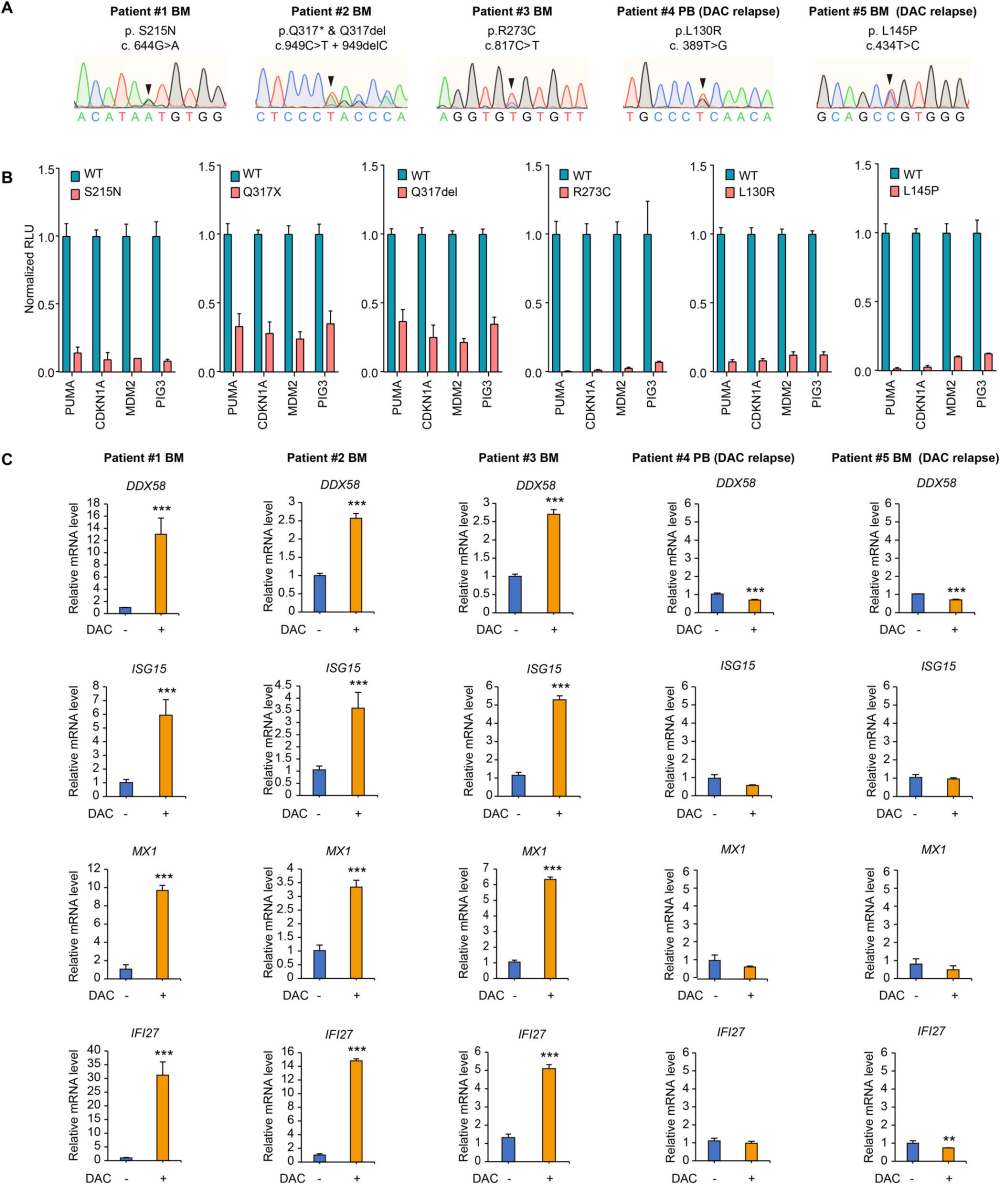
Decitabine (DAC) failed to effectively activate interferon (IFN) signaling in the primary myeloid malignant cells derived from acute myeloid leukemia/myelodysplastic syndrome (AML/MDS) patients with DAC relapse. (A) *TP53* Sanger sequencing results of the primary bone marrow mononuclear cells (BMMCs) and peripheral blood mononuclear cells (PBMCs) derived from the five indicated AML/MDS patients. (B) Luciferase reporter assays for the six p53 mutants derived from the five AML/MDS patients. Wild‐type (WT) p53 was served as control. (C) qRT‐PCR determination of the indicated genes in the indicated primary BMMCs and PBMCs upon 0.2 µM DAC treatment for 3 days. Error bars represent mean ± SD (*n* = 3, *p < 0.05, **p < 0.01, ***p < 0.001, ****p < 0.0001)

In summary, we report the activation of IFN signaling in DAC‐treated p53‐deficient myeloid malignant cells and the failure of DAC in activating IFN signaling in resistant cells.

## CONFLICT OF INTEREST

The authors declare no competing interests.

## Supporting information

Supporting information

figureS1

figureS2

figureS3

figureS4

figureS5
