# Supplementary material for: Decitabine activates type I interferon signaling to inhibit p53‐deficient myeloid malignant cells
Source: Clin Transl Med. 2021 Nov 6;11(11):e593. doi: 10.1002/ctm2.593 (PMC8571953; doi:10.1002/ctm2.593)
Supplement: Supplementary file 1 — Supporting information [file CTM2-11-e593-s005.docx]

**1 SUPPLEMENTAL FIGURE LEGENDS**

**Figure S1.** Clinical implication of *TP53* mutation in myeloid malignance. (**A**) Log-rank test in univariate Cox proportional hazard model for the ten TCGA cancer types with the highest HRs for *TP53* mutation. Cohorts with > 100 patients were selected for presentation. (**B**) Log-rank test in univariate Cox proportional hazard model for the most frequent gene mutations in the TCGA AML cohort. (**C**) Kaplan–Meier survival curves shows HRs and *P* values (Log-rank test in univariate Cox proportional hazards model) of *TP53* mutation in the TCGA AML cohort. (**D**) The reported HRs of *TP53* mutation in AML/MDS patients ^[1-5]^.

**Figure S2.** DAC preferentially inhibits p53-deficient MEFs and isogenic THP-1 cell lines. (**A**) MEFs of the indicated genotypes prepared from different embryos of a same litter were treated with DAC for 3 days, followed by cell viability determination. (**B**) qRT-PCR determination of the indicated genes in MEFs upon 1 μM DAC treatment for 3 days. (**C**) Immunofluorescence of THP-1 cells expressing different status of p53. p53 were stained by DO1. (**D**) THP-1 cells were treated with DAC for 3 days, followed by cell viability determination. (**E**) Heatmap shown relative expression levels of type I IFN signaling genes in the DAC-treated U937 expressing p53-R282W cells. Error bars represent mean ± S.D. (n = 3, * *P* < 0.05, ** *P* < 0.01).

**Figure S3.** Preparation of DAC-resistant THP-1 cells. **(A)** The schematic presentation of the process of establishing DAC-resistant cell line from parental THP-1 cell line. (**B**) Representative images of the indicated cells upon 1 μM DAC treatment for 0-6 days.

**Figure S4.** DAC activates IFN signaling in p53-deficient AML mouse model. (**A**) Spleen and liver weights of moribund AML and age-matched normal mice. (**B**) Complete blood counts assay from moribund AML and age-matched normal mice. (**C-E**) Representative flow cytometry profiles of BM, PB, and SPs derived from the indicated mice. The markers for myeloid cells (**C**), lymphoid cells (**D**), T-cells (**D**), and stem cell progenitors (**E**) were used. * *P* < 0.05, ** *P* < 0.01.

**Figure S5.** Cell viability analysis of cultured PMBC/BMMC *in vitro*. **(A)** Four PBMC/BMMC samples were thawed and cultured in RPMI 1640 medium for 3 days. Cell viability was analyzed by trypan blue exclusion test. Total and viable cell number was quantified using automatic cell counter (BodBoge, JSY-SC-031N, China). (**B**) Representative images of fresh thawed and cultured PBMC sample 1. Red arrow indicates dead cell, scare bar 50 μm.

**2 MATERIALS AND METHODS**

**2.1** **TCGA Data mining**

To analyze gene mutation-associated overall survival of cancer patients in the TCGA, gene mutation data and survival data were downloaded from cBioPortal (<http://www.cbioportal.org/>) for analysis. Thirty-two cancer cohorts labeled with “TCGA provisional” were selected for analysis. Cohorts with patient numbers < 100 or *TP53* mutation rate < 5% were further excluded from analysis. HNSC, LIHC, PAAD, BLCA, BRCA, LUAD, OV, PRAD, and UCEC are short for Head and Neck Squamous Cell Carcinoma, Liver Hepatocellular Carcinoma, Pancreatic Adenocarcinoma, Bladder Urothelial Carcinoma, Breast Invasive Carcinoma, Lung Adenocarcinoma, Ovarian Serous Cystadenocarcinoma, Prostate Adenocarcinoma, and Uterine Corpus Endometrioid Carcinoma, respectively.

**2.2 Cell cultures**

Leukemia cell lines THP-1 and U937 were obtained from ATCC. THP-1 and engineered cells were cultured in RPMI 1640 Medium (Gibco) with 10% fetal bovine serum (FBS, Moregate, Australia), 100 U/ml penicillin, and 100 mg/ml streptomycin. Bone marrow (BM), peripheral blood (PB) cells and feal liver cells from moribund mice or AML/MDS patient were cultured in IMDM (Gibco) or RMPI 1640 medium with 20% FBS, 100 U/ml penicillin, and 100 mg/ml streptomycin. Cell cultures were maintained in a 5% CO_2_ humidified incubator at 37 °C. All cell lines were confirmed to be mycoplasma-free.

**2.3 Immunostaining**

Cells were fixed in 4% phosphate-buffered saline (PBS)–paraformaldehyde for 15 min, incubated in 0.2% Triton X-100 for 5 min, then 0.2% fish skin gelatin in PBS for 10 min, and stained for 1 h with primary antibody. Staining with secondary antibody was performed for 20 min, followed by three washes and visualization under a fluorescence confocal microscope.

**2.4 Cell viability assay**

Cell viability was assayed using a Cell Counting Kit-8 (A311-02, Vazyme Biotech, China) in accordance with the manufacturer’s instructions.

**2.5 RNA-seq**

THP-1 or U937 cells infected with p53-R282W or WT p53 were treated with 1 or 5 μM decitabine (DAC, Target Mol, # T1508) for 2 days. Total RNA was isolated using a total RNA Purification Kit (B518651, Sangon Biotech). Sequencing libraries were constructed from 1 μg of purified mRNA using an Illumina TruSeq RNA Sample Prep Kit (cat# FC-122-1001). Libraries were pooled and 150-bp paired-end reads were sequenced on the Illumina HiSeq Xten platform. For bioinformatics analyses, raw sequence reads were initially processed using FastQC (Babraham Institute, Cambridge, UK) for quality control, then adapter sequences and poor-quality reads were removed using Cutadapt. Quality-filtered reads were then mapped to the human genome (hg38) using STAR software, and only uniquely mapped reads were kept. Read counts were calculated using the GFOLD package. THP-1 native and DAC-resistant cells were treated with 1 μM DAC for 6 days. Total RNA was isolated using a total RNA Purification Kit (B518651, Sangon Biotech). RNA was evaluated for quantity and quality for a minimum RIN score of 7 or higher using Agilent Bioanalyzer 2100. cDNA libraries were prepared using RNA fragmentation, cDNA synthesis, ligation of index adaptors, and amplification using KAPA mRNA HyperPrep Kit (KK8581). RNA-seq was conducted on the Illumina NovaSeq 6000 platform according to the manufacturer's instructions. Raw sequence reads were initially processed using FastQC (Babraham Institute, Cambridge, UK) for quality control, then adapter sequences and poor-quality reads were removed using Cutadapt ((v1.9.1)). Clean data were aligned to reference human genome (hg38, downloaded from Ensembl browser) via software Hisat2 (v2.1.0) and ordered by samtools (v1.6). Then HTSeq (v0.13.5) were used to estimate gene and repeat element expression levels using different reference files. Gene reference files was GRCh38.gtf downloaded from Ensembl browser, and repeat element reference files was hg38_rmsk_TE.gtf integrated by MHammell Lab ^[6]^. To discovery differentially expressed dsRNAs, we analyzed the raw counts with R package DESeq2 (v 1.28.1). Repeat elements with log2(fold change) >1 and *P* < 0.05 were considered as differentially expressed in either DAC-treated native cells vs untreated native cells or in DAC-treated DAC-resistant cells vs untreated DAC-resistant cells. Next, heatmap were generated using the R with pheatmap (v1.0.12).

**2.6 Protein interaction network**

Protein interaction networks were analyzed via the STRING database (https://string-db.org/, version 10.5) by inputting the 216 upregulated genes for p53-R282W cells and 332 upregulated genes for p53 wild type cells with the minimum required interaction score setting using the highest confidence (0.900).

**2.7 Enrichment assay**

Enrichment assays were performed using Enrichr (https://maayanlab.cloud/Enrichr/) by inputting the 216 upregulated genes. Analysis of the expression data was performed using Gene set enrichment analysis (GSEA) implemented in java GSEA application, version 2.0.

**2.8 Real-time qRT-PCR**

Total RNA was isolated from cells using a Total RNA Purification Kit (B518651, Sangon Biotech), then 1 μg total RNA was reverse-transcribed using the HiScript^®^ II Q RT SuperMix for qPCR (+ gDNA wiper) (R223-01, Vazyme Biotech, China) following the manufacturer’s protocol. PCR was performed in triplicate using ChamQ^TM^ SYBR^®^ qPCR Master Mix (Low ROX Premixed) (Q331-02/03, Vazyme Biotech, China) and a ViiA™ 7 Real-Time PCR System (Applied Biosystems) under the following conditions: 5 min at 95 °C, followed by 40 cycles of 95 °C for 15 s and 60 °C for 60 s. Specificities of PCR products were checked for each primer set and sample by melting curve analysis. Expression levels of genes were normalized relative to levels of β-actin adopting the comparative Ct method. The primers for human genes: *DDX58* F: 5′-ATCCC-AGTGTATGAACAGCAG-3′, R: 5′-GCCTGTAACTCTATACCCATGTC-3′; *ISG15* F: 5′-GGACAAATGCGACGAACCTCT-3′, R: 5′-GCCCGCTCACTTGCTGCTT-3′; *MX1* F: 5′-ttcagcacctgatggccta-3′, R: 5′-tggatgatcaaagggatgtg-3′; *IFI27* F: 5′-ACCTCATCAGCAGTGACCAGT-3′, R: 5′-ACATCATCTTGGCTGC-TATGG-3′; *MER57B1*-F: CCTCCTGAGCCAGAGTAGGT; R: ACCAGTCTGGCTG-TTTCTGT; ERV3-F: AGCAGCCCAGGACAAGT; R: CGACATCCTTGCCAGAGG-G and *β-actin* F:5′-ACTTAGTTGCGTTACACCCTTTCT-3′, R: 5′-GACTGCTGTC-ACCTTCACCGT-3′. Primers for mouse genes: *Ddx58* F: 5′-caaaccgggcaacaggaatg-3′, R: 5′-atctccgctggctctgaatg-3′; *Isg15* F: 5′-cctctgagcatcctggt-3′, R: 5′-aggccgtactcccccag-3′; *Irf7* F: 5′-ggtgtgtccccaggatcattt-3′, R: 5′-gcatagggttcctcgtaaaca-3′; *Ifi27* F: 5′-catcattggattcggttcctgt-3′, R: 5′-ccttcttgctgctttgcctg-3′; *Ifi44* F: 5′-agtcctgtgaagtccaagctg-3′, R: 5′-cagctgccactctgagacat-3′; and *β-actin* 5′-ggctgtattcccctccatcg-3′, 5′-ccagttggtaacaatgccatgt-3′.

**2.9 Establishment of DAC resistant THP-1 cell line**

To prepare the DAC-resistant cell line, THP-1 cells were treated with the IC_90_ dose of DAC until 90% cell death was reached, then cultured in DAC-free medium until confluence, followed by another round of DAC treatment. The treatment cycles were repeated for 3 months, until significant DAC resistance was observed.

**2.10 Methylation-specific PCR**

THP-1 and THP-1 DAC-resistant cells were treated with 0.2 or 0.5 μM DAC for 6 days. Genomic DNA was extracted using a Blood Genomic DNA Mini Kit (CW2087M, cwbiotech, China). Genomic DNA was bisulfite modified by EZ DNA methylation-gold kit (Zymo Research, Orange, CA, USA). Methylation-specific PCR primers were designed according to genomic sequences flanking the presumed transcription start sites for *IFNB1*, *IFNA1*, and *IRF7*. Primer sequences were oligo-synthesized (IDT) to allow methylation specific PCR to detect bisulfite-induced changes affecting unmethylated (U) and methylated (M) alleles. Methylation specific PCR primers for *IFNB1*, *IFNA1*, and *IRF7* are as follows: *IFNB1*-M-F: TTGTTTTGGTATAATAGGT-AGTAGGC; *IFNB1*-M-R: TCTCATAAATAATCAATACGACGTC; *IFNB1*-U-F: TT-TTGGTATAATAGGTAGTAGGTGA; *IFNB1*-U-R: TCTCATAAATAATCAATACA-ACATC; *IFNA1*-M-F: GGTTGGAGTGTAGTGGTATAATTTC; *IFNA1*-M-R: ATCC-CAACACTTTAAAAATCCG; *IFNA1*-U-F: AGGTTGGAGTGTAGTGGTATAATTT-T; *IFNA1*-U-R: ATAATCCCAACACTTTAAAAATCCA; *IRF7*-M-F: TAGGTGTAG-TACGTAGATAGACGGC ;*IRF7*-M-R: CCCAACTTACAAATAAAAAAACGAC *IRF7*-U-F: GGTGTAGTATGTAGATAGATGGTGG; and *IRF7*-U-R: CCAACTTAC-AAATAAAAAAACAAC. Each methylation-specific PCR reaction incorporated ~200 ng of bisulfite treated DNA as template, 400 nM of each primer, 2 × Taq Master Mix (Dye Plus) (P112-01, Vazyme, China) in a final reaction volume of 10 μL. Cycle conditions were as follows: 95°C, 5 minutes; 40 cycles, (95°C, 20 seconds, 50°C, 15 seconds, and 72°C, 1 minute); and 72°C, 5 minutes. Methylation-specific PCR products were analyzed with 1.5% agarose gel electrophoresis and stained with YeaRed dye (Yeasen, 10202ES76, China).

**2.11 Flow cytometry analysis of hematopoietic cells and tissues**

The stained cells were analyzed on a BD LSRFortessa™ X-20 flow cytometer (BD Biosciences). Antibodies specific for surface antigens (B220 (RA3-6B2), CD19 (eBio1D3), Mac-1 (M1/70), Gr-1 (RB6-8C5), CD3 (145-2C11), Sca-1 (D7), TER119 (TER-119), CD71 (C2), CD34 (RAM34), cKit (2B8), and CD48 (HM48-1)) were purchased from eBioscience; Lineage markers (559971, Mac-1, Gr-1, CD3, B220, and Ter119) were purchased from BD Biosciences. CD150 (TC15-12F12.2) and CD16/32 (93) were purchased from Biolegend. Lin^-^CD48^-^cKit^+^Sac1^+^CD150^+^ cells were defined as HSCs, Lin^-^CD48^-^cKit^+^Sac1^+^CD150^-^ cells were defined as multipotential progenitors (MPPs), Lin^-^cKit^+^Sac1^-^ cells were defined as MPs, Lin^-^cKit^+^Sac1^-^CD34^high^CD16/32^low^ cells were defined as CMPs, Lin^-^cKit^+^Sac1^-^CD34^high^ CD16/32^high^ cells were defined as GMPs, and Lin^-^cKit^+^Sac1^-^CD34^low^ CD16/32^low^ cells were defined as MEPs.

**2.12 Mouse model construction**

*Trp53*^-/-^ mice (jax: 002101) were bred in the Ruijin Hospital animal experiment center. Genotyping was performed according to standard protocols available at https://www.jax.org/. All mice were on a C57BL/6 background. To construct the AML mouse model, hematopoietic stem and progenitor cells (HSPCs) were isolated from fetal livers (embryonic day 13.5), cultured, and retrovirally transduced with GFP-IRES-*Kras*^G12D^ constructs according to the method of Schmitt et al. ^[7]^. Approximately 2 × 10^6^ HSPCs were then injected into the tail veins of 6–8-week-old lethally irradiated syngeneic recipient mice. For secondary leukemia transplantation, 2 × 10^6^ leukemia cells freshly harvested from bone marrow were transplanted into sub-lethally irradiated syngeneic recipient mice.

**2.13 Mouse treatment**

For DAC treatment, mice were i.v. injected with 2 × 10^6^ AML cells isolated from 1^st^-transplantation mice. After 3 days, mice were i.p. injected with 0.2 mg kg^-1^ DAC or PBS for 5 consecutive days per week. Mouse survival was monitored every day. All animal work complied with Ruijin Hospital animal ethical regulations. The Shanghai Institute of Hematology approved the relevant study protocols. Experiments were carried out according to the National Institutes of Health Guide for the Care and Use of Laboratory Animals.

**2.14 *TP53* status determination**

Primary human AML/MDS PB or BM samples were obtained from Shanghai Institute of Hematology of Ruijin Hospital. Genomic DNA was extracted using a Blood Genomic DNA Mini Kit (CW2087M, cwbiotech, China). PCR was performed with specific primers and then subjected to Sanger sequence. The primers for PCR: PCR-*TP53*-(2-3)-F: CCGAGCTGTCTCAGACACT; PCR-*TP53*-(2-3)-R: AGAGCA- GTCAGAGGACCAG; PCR-*TP53*-(4-6)-F: ACCTGTGGGAAGCGAAAATT; PCR- *TP53*-(4-6)-R: ACTGTGCAATAGTTAAACCC; PCR-*TP53*-(7-9)-F: CCTCATCTTG- GGCCTGTGTT; PCR-*TP53*-(7-9)-R: TAGCTACAACCAGGAGCCAT; PCR-*TP53*- (10-11)-F: ATGTTGCTTTTGTACCGTCAT; PCR-*TP53*-(10-11)-R: TCCCCACAA- CAAAACACCAG. The primers for Sanger sequencing: Seq-*TP53*-(exon 2-3): CCGAGCTGTCTCAGACACT; Seq-*TP53*-(exon 4): ACAGGAGTCAGAGATCAC- AC; Seq-*TP53*-(exon 5-6): TCCTGAGGTGTAGACGCCAA; Seq-*TP53*-(7): TGGA- AGAAATCGGTAAGAGG; Seq-*TP53*-(8-9): GGCGGGGAATCTCCTTACTG; Seq- *TP53*-(10): ATGTTGCTTTTGTACCGTCAT; Seq-*TP53*-(11): CTTAGGCCCTTCA- AAGCATT. The study was approved by the Review Boards of Ruijin Hospital and informed consent was obtained in accordance with the Declaration of Helsinki.

**2.15 Luciferase reporter assay**

Luciferase reporter assay was performed as reported previously ^[8]^. Briefly, cells were seeded in 100 µL medium at 30% density in 96-well plates for 24 h. To do the transfection for 10 wells, 3 µL FuGENE transfection reagent (Promega, E1960) and 50 µL Opti-MEM were mixed and incubated for 5 min, and then added to the pre-mixed three plasmids (750 ng p53-expressing plasmid, 250 ng of luciferase re-porter plasmid, 12 ng of Renilla plasmid). Upon thorough mix, they were incubated for 15 min at room temperature. The mixture was then added to 1 mL room-temperature DMEM medium and thoroughly mixed. The ~1 mL mixture were then used to replace all the culturing medium in 10 wells (100 µL per well). After 24 h, cells were lysed, followed by luciferase signal determination using a luciferase assay kit (Vazyme, DL101-01). The luminescent signal of firefly luciferase was normalized against that of Renilla luciferase for each sample.

**2.16 Statistics**

Kaplan–Meier survival analysis was performed and survival differences between groups were assessed with the Log-rank test using SPSS 24.0 version. Unpaired two-tailed Student’s *t*-tests were used to determine the significance between two data sets, assuming significance at * *P* < 0.05, ** *P* < 0.01, *** *P* < 0.001, **** *P* < 0.0001, *n.s*., not significant.

**References**

1. Parkin, B., H. Erba, P. Ouillette, et al. (2010). Acquired genomic copy number aberrations and survival in adult acute myelogenous leukemia. *Blood*, 116(23), 4958-4967.

2. Parkin, B., P. Ouillette, M. Yildiz, K. Saiya-Cork, K. Shedden, and S.N. Malek. (2015). Integrated genomic profiling, therapy response, and survival in adult acute myelogenous leukemia. *Clin Cancer Res*, 21(9), 2045-2056.

3. Kulasekararaj, A.G., A.E. Smith, S.A. Mian, et al. (2013). TP53 mutations in myelodysplastic syndrome are strongly correlated with aberrations of chromosome 5, and correlate with adverse prognosis. *Br J Haematol*, 160(5), 660-672.

4. Bejar, R., K. Stevenson, O. Abdel-Wahab, et al. (2011). Clinical effect of point mutations in myelodysplastic syndromes. *N Engl J Med*, 364(26), 2496-2506.

5. Ok, C.Y., K.P. Patel, G. Garcia-Manero, et al. (2015). TP53 mutation characteristics in therapy-related myelodysplastic syndromes and acute myeloid leukemia is similar to de novo diseases. *J Hematol Oncol*, 8, 45.

6. Jin, Y., O.H. Tam, E. Paniagua, and M. Hammell. (2015). TEtranscripts: a package for including transposable elements in differential expression analysis of RNA-seq datasets. *Bioinformatics*, 31(22), 3593-3599.

7. Schmitt, C.A., J.S. Fridman, M. Yang, E. Baranov, R.M. Hoffman, and S.W. Lowe. (2002). Dissecting p53 tumor suppressor functions in vivo. *Cancer Cell*, 1(3), 289-298.

8. Chen, S., J.L. Wu, Y. Liang, et al. (2021). Arsenic Trioxide Rescues Structural p53 Mutations through a Cryptic Allosteric Site. *Cancer Cell*, 39(2), 225-239 e228.
