# Supplementary figures and images for: Decitabine activates type I interferon signaling to inhibit p53‐deficient myeloid malignant cells
Source: Clin Transl Med. 2021 Nov 6;11(11):e593. doi: 10.1002/ctm2.593 (PMC8571953; doi:10.1002/ctm2.593)

**Figure S1**

**A**

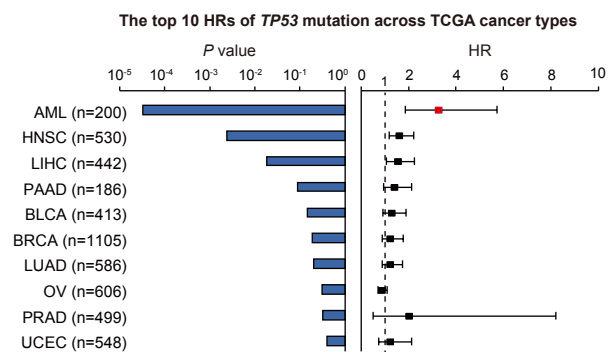

**B**

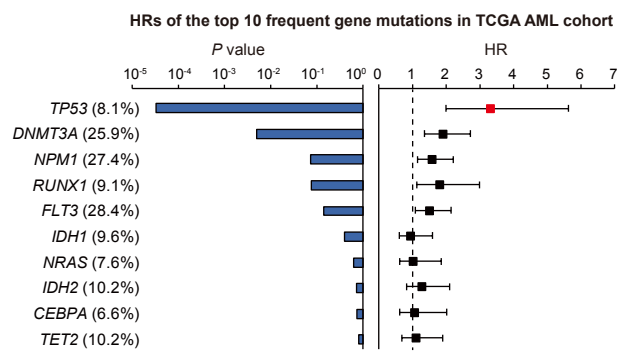

**C**

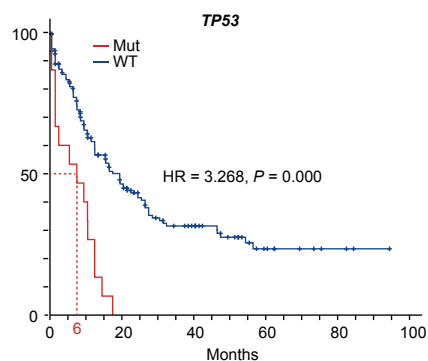

**D**

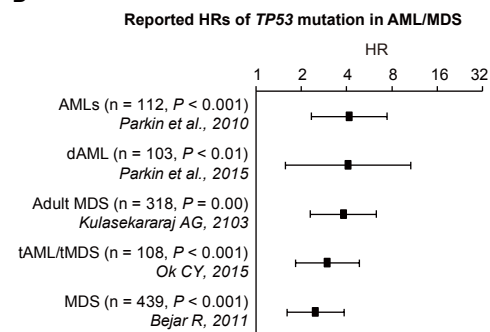

Supplement: Supplementary file 2 — figureS1 [file CTM2-11-e593-s003.pdf]

Figure S2

A

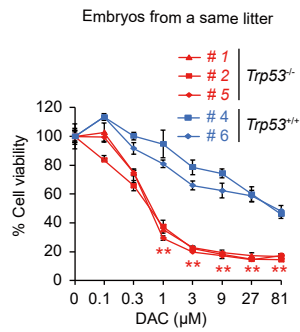

B

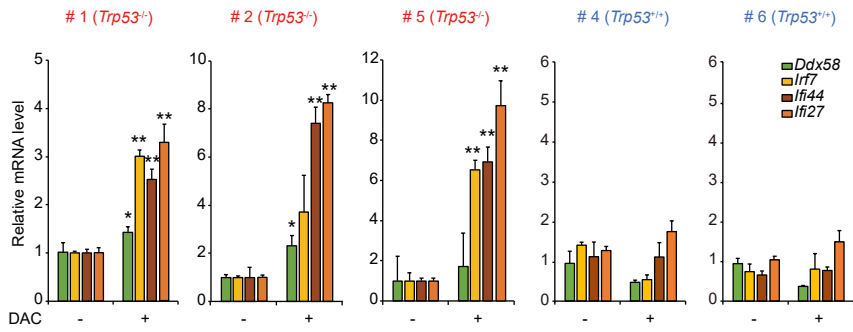

C

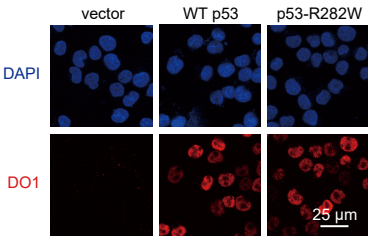

D

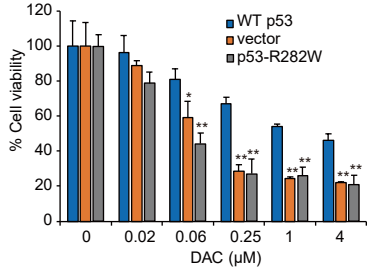

E

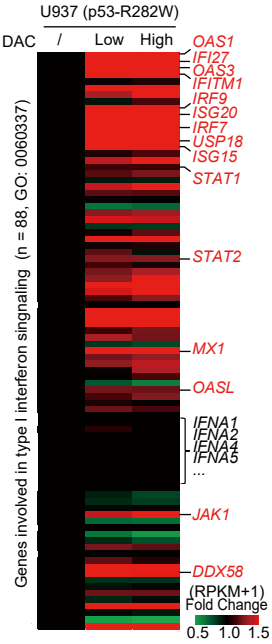

Supplement: Supplementary file 3 — figureS2 [file CTM2-11-e593-s006.pdf]

Figure S3

A

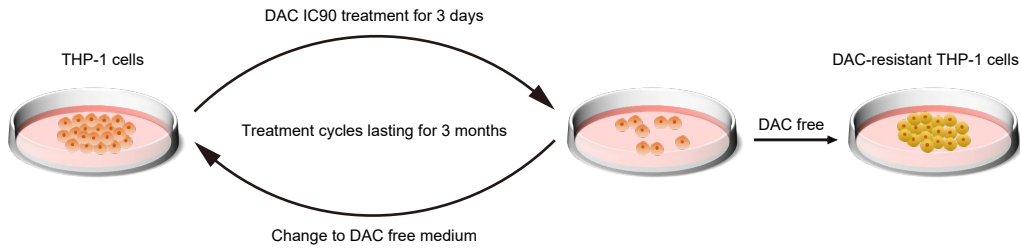

B

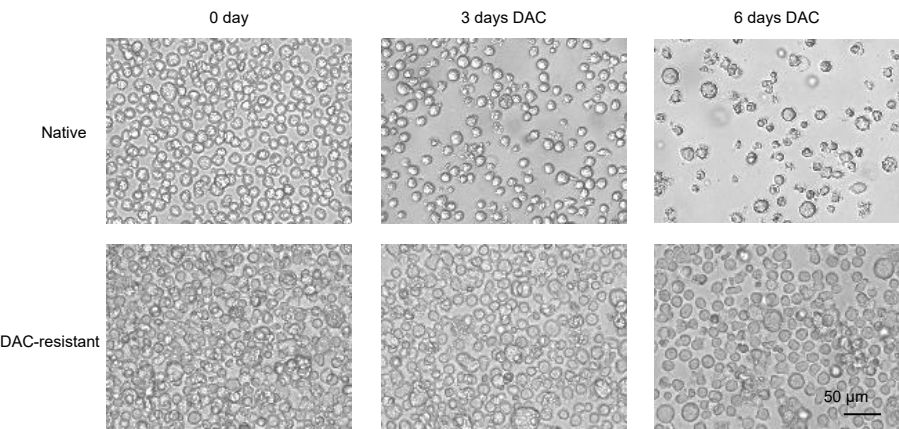

Supplement: Supplementary file 4 — figureS3 [file CTM2-11-e593-s002.pdf]

**Figure S4**

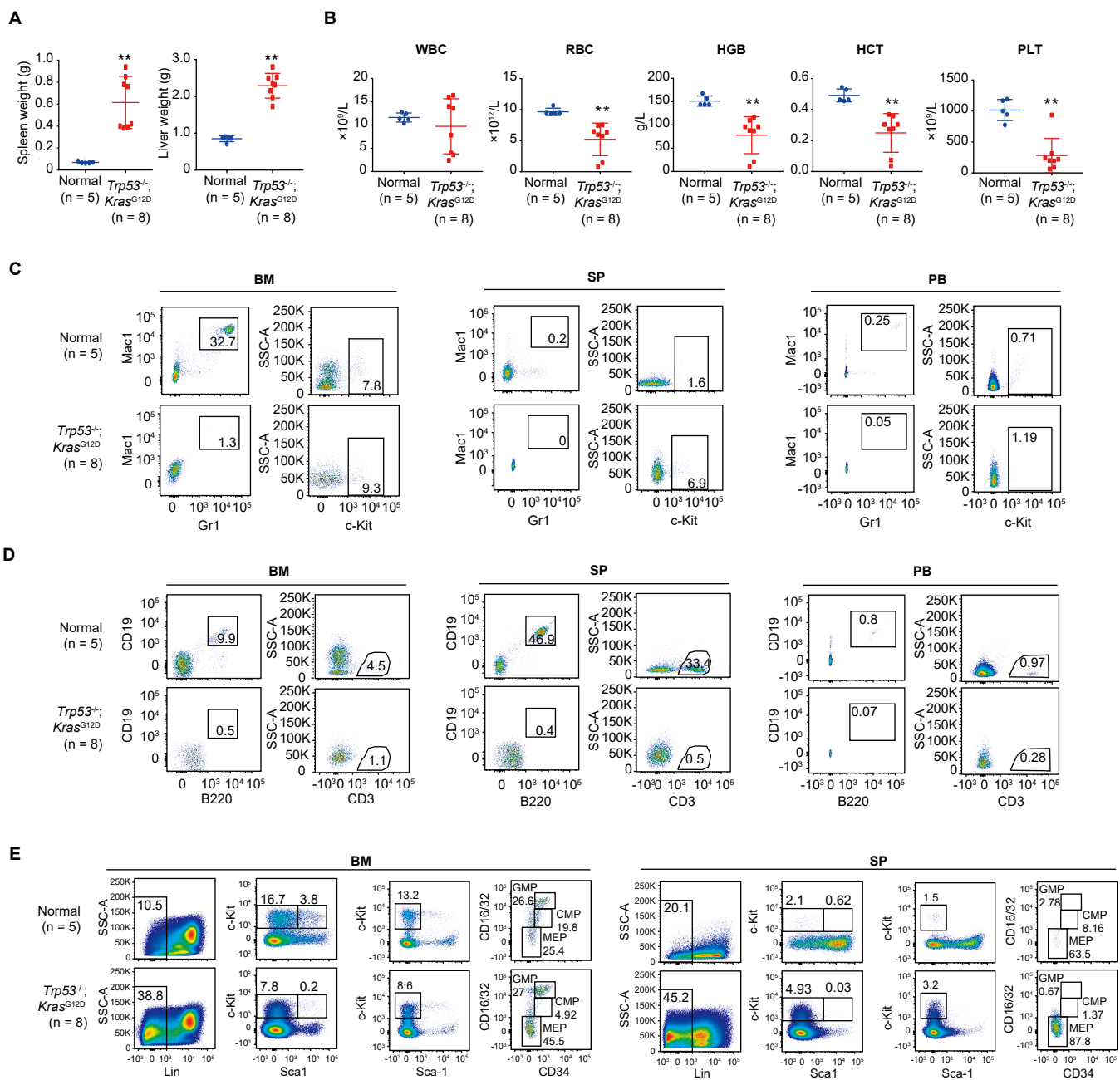

Supplement: Supplementary file 5 — figureS4 [file CTM2-11-e593-s001.pdf]
