# Supplementary material for: Decitabine activates type I interferon signaling to inhibit p53‐deficient myeloid malignant cells
Source: Clin Transl Med. 2021 Nov 6;11(11):e593. doi: 10.1002/ctm2.593 (PMC8571953; doi:10.1002/ctm2.593)

Figure S5

A

In vitro cultured PBMC/BMMC cells

days / counts / sample no.	0 day			3 days		
	viable (x 10^6/mL)	total (x 10^6/mL)	viable/total	viable (x 10^6/mL)	total (x 10^6/mL)	viable/total
1 (PBMC)	82.9	84.4	98%	75.1	80.7	93%
2 (BMMC)	67.1	69.9	96%	62.9	69.1	91%
3 (BMMC)	67.9	70.9	96%	59.3	64.7	92%
4 (PBMC)	89.7	92.4	97%	70.4	75.7	93%

B

Sample 1, day 0

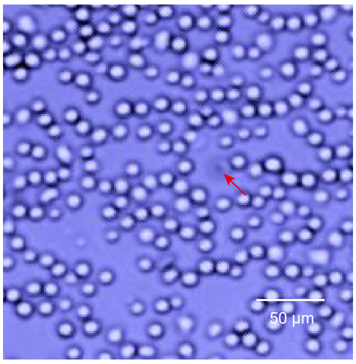

Sample 1, day 3

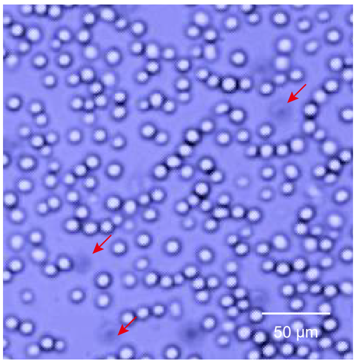

Supplement: Supplementary file 6 — figureS5 [file CTM2-11-e593-s004.pdf]
